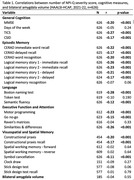# Exploring the relationship between neuropsychiatric symptoms, cognitive test performance, and brain abnormalities in a cohort of older adults in rural South Africa

**DOI:** 10.1002/alz.092788

**Published:** 2025-01-03

**Authors:** Sarah Gao, Darina T. Bassil, Meagan T. Farrell, Ryan G. Wagner, Jennifer J. Manly, Adam M. Brickman, Stephen Tollman, Lisa F. Berkman

**Affiliations:** ^1^ Harvard T. H. Chan School of Public Health, Cambridge, MA USA; ^2^ Harvard T.H. Chan School of Public Health, Cambridge, MA USA; ^3^ IQVIA, New York, NY USA; ^4^ University of the Witwatersrand, Johannesburg South Africa; ^5^ Taub Institute for Research on Alzheimer’s Disease and the Aging Brain, New York, NY USA; ^6^ Department of Neurology, Vagelos College of Physicians and Surgeons, Columbia University, and the New York Presbyterian Hospital, New York, NY USA; ^7^ Taub Institute for Research on Alzheimer’s Disease and the Aging Brain, Columbia University, New York, NY USA; ^8^ The Gertrude H. Sergievsky Center, College of Physicians and Surgeons, Columbia University, New York, NY USA; ^9^ Department of Neurology, Vagelos College of Physicians and Surgeons, Columbia University, New York Presbyterian Hospital, New York, NY USA; ^10^ Department of Neurology, Columbia University, New York, NY USA; ^11^ Department of Neurology, College of Physicians and Surgeons, Columbia University, New York, NY USA; ^12^ Columbia University, New York, NY USA; ^13^ Department of Neurology, Columbia University Irving Medical Center, New York, NY USA

## Abstract

**Background:**

People with Alzheimer’s Disease (AD) often have neuropsychiatric symptoms. The Neuropsychiatric Inventory (NPI) is a validated tool for assessing the severity of these symptoms, but its applicability in a South African context remains unexamined. This study evaluated NPI‐Q, a brief version of the NPI, in relation to cognitive performance and markers of brain atrophy and emotional regulation among older South African adults.

**Method:**

Data were from Cognition and Dementia in the Health and Aging in Africa: A Longitudinal Study in South Africa (HAALSI‐HCAP), a population‐based nested cohort study in Agincourt, South Africa. The survey included a cognitive battery, clinical examination, and informant interview to assess perceived cognitive and functional changes in respondents. During the informant interview, the NPI‐Q was completed, and a severity score was derived from the cumulative sum of reported individual symptom scores (range: 0‐36). The final sample included 626 participants (71.9±11.3 years‐old, 35.6% men, 56.6% without formal education), of whom 185 underwent MRI scans. Pairwise correlations assessed the relationship between informant reported symptom severity with cognitive measures and MRI‐generated bilateral amygdala volume.

**Result:**

A total of 95 informants (15.2%) reported any neuropsychiatric symptoms, averaging 2.48 per participant among those who reported any symptom. Hallucinations were the most common symptom (5.4%), while disinhibition was the least (1%). Among those who reported symptoms, average symptom severity score was 4.28 (SD: 3.65). Severity was significantly correlated (p‐value<0.001) with most cognitive measures assessing general cognition (β: ‐0.27, ‐0.17), episodic memory (β: ‐0.26, ‐0.17), language (β: ‐0.28, ‐0.12), and executive function (β: ‐0.26, ‐0.12). However, no relationship was observed between neuropsychiatric symptoms and visuospatial functioning or amygdala volume, possibly attributable to high rate of missingness for visuospatial measures (30%), and the small MRI sample size reporting symptoms (n = 26).

**Conclusion:**

NPI‐Q symptoms were associated with lower cognitive performance, revealing a significant relationship between symptom severity and cognitive performance across all measures of the HAALSI‐HCAP cognitive battery, except for those evaluating visuospatial function. Associations between neuropsychiatric symptoms and bilateral amygdala volume were less evident. These results contribute to understanding the impact of neuropsychiatric symptoms on cognitive health in aging South African populations.